# Protein tyrosine kinase, PtkA, is required for *Mycobacterium tuberculosis* growth in macrophages

**DOI:** 10.1038/s41598-017-18547-9

**Published:** 2018-01-09

**Authors:** Dennis Wong, Wu Li, Joseph D. Chao, Peifu Zhou, Gagandeep Narula, Clement Tsui, Mary Ko, Jianping Xie, Carlos Martinez-Frailes, Yossef Av-Gay

**Affiliations:** Division of Infectious Diseases, Departments of Medicine and Microbiology and Immunology, University of British Columbia, Life Sciences Institute, Vancouver, British Columbia V6T-1Z3 Canada

## Abstract

Protein phosphorylation plays a key role in *Mycobacterium tuberculosis* (Mtb) physiology and pathogenesis. We have previously shown that a secreted protein tyrosine phosphatase, PtpA, is essential for Mtb inhibition of host macrophage acidification and maturation, and is a substrate of the protein tyrosine kinase, PtkA, encoded in the same operon. In this study, we constructed a ∆*ptkA* deletion mutant in Mtb and found that the mutant exhibited impaired intracellular survival in the THP-1 macrophage infection model, correlated with the strain’s inability to inhibit macrophage phagosome acidification. By contrast, the mutant displayed increased resistance to oxidative stress *in vitro*. Proteomic and transcriptional analyses revealed upregulation of *ptpA*, and increased secretion of TrxB2, in the Δ*ptkA* mutant. Kinase and protein-protein interaction studies demonstrated that TrxB2 is a substrate of PtkA phosphorylation. Taken together these studies establish a central role for the *ptk*A-*ptp*A operon in Mtb pathogenesis.

## Introduction

The etiological agent of tuberculosis (TB), *Mycobacterium tuberculosis* (Mtb), remains one of the most devastating infectious agents in the world. One-third of the world’s population is exposed to Mtb, resulting in nearly two million deaths annually^1^. The emergence of extensively drug-resistant (XDR) strains and co-infection with HIV has exacerbated the global burden of TB^2^. Therefore, there is an urgent need for the development of novel therapeutics against the deadly disease.

Mtb primarily infects alveolar macrophages, the first line of defense against microbial invasion in the lung. Macrophages engulf the bacteria into phagosomes which acidify and mature by remodeling the membrane and luminal contents^3^. Antimicrobial molecules such as acidic hydrolases are delivered to the compartment, and interaction with the endosomal network ultimately leads to phagosome-lysosome fusion and elimination of the foreign particle^4^. However, Mtb blocks phagosome maturation and prevents the phagosome from acquiring antimicrobial properties, enabling its survival within the hostile environment of the macrophage^5–8^.

Mtb utilizes multiple mechanisms to protect itself from the host antimicrobial assault. The Mtb cell wall glycolipid ManLAM co-operates with the secreted acid phosphatase SapM to block phagosome fusion with late endosomes. Both prevent phagosomal accumulation of phosphatidylinositol 3-phosphate (PI3P), which mediates the recruitment of membrane trafficking proteins such as the early endosomal antigen, EEA1^9,10^. Mtb is resistant to host-generated reactive oxygen and nitrogen species; ROS and RNS are adsorbed in its thick cell wall and inactivated by detoxification enzymes or antioxidants, such as catalase (KatG)^11^, peroxidase and the peroxynitrite reductase complex (AhpC, AhpD, DlaT and Lpd)^12^, the thioredoxin system^13^, and mycothiol^14^.

Mtb has evolved systems to sense the stages of engulfment within macrophages and activate bacterial systems to block host defense pathways^15–17^. Mtb possesses a wide repertoire of signal transduction systems, including eleven “two-component” systems, eleven eukaryotic-like serine/threonine protein kinases (PknA-PknL), two protein tyrosine phosphatases (PtpA and PtpB), and the newly identified protein tyrosine kinase, PtkA^8,18–22^. These signaling proteins play key roles in bacterial adaptation and response to host defense mechanisms. In particular, PtpA, a secreted protein essential for Mtb pathogenesis, interacts with the host vacuolar-H^+^-ATPase complex to block its trafficking to the mycobacterial phagosome, thereby preventing phagosome acidification and ultimately phagosome-lysosome fusion^15,20^. PtkA, encoded within the same operon as *ptpA*, phosphorylates PtpA^19^, thereby enhancing PtpA phosphatase activity^23^. This suggests that PtkA and PtpA may act cooperatively to provide a function essential for Mtb pathogenesis.

In this work, we constructed a deletion mutant of *ptkA* to assess its role in Mtb physiology. The Δ*ptkA* strain was found to have impaired intracellular survival within the THP-1 macrophage infection model, and failed to inhibit phagosome acidification. However, the Δ*ptkA* mutant strain also displayed enhanced resistance against oxidative stress *in vitro*. We therefore used a global proteomics approach to compare Δ*ptkA* and its parental wild type (WT) strain when exposed to oxidative stress. Additionally, given that PtpA is secreted, we compared the secretomes of Δ*ptkA* and its parental strain. We discovered that thioredoxin reductase TrxB2, an important protein in the defense against oxidative stress, is secreted by Mtb and its secretion is up-regulated in the Δ*ptkA* mutant strain. Through kinase assays and protein-protein interaction studies, we discovered that TrxB2 is a substrate of PtkA phosphorylation.

## Results

### Construction of a *ptkA* knockout mutant in Mtb

**Figure 1:**
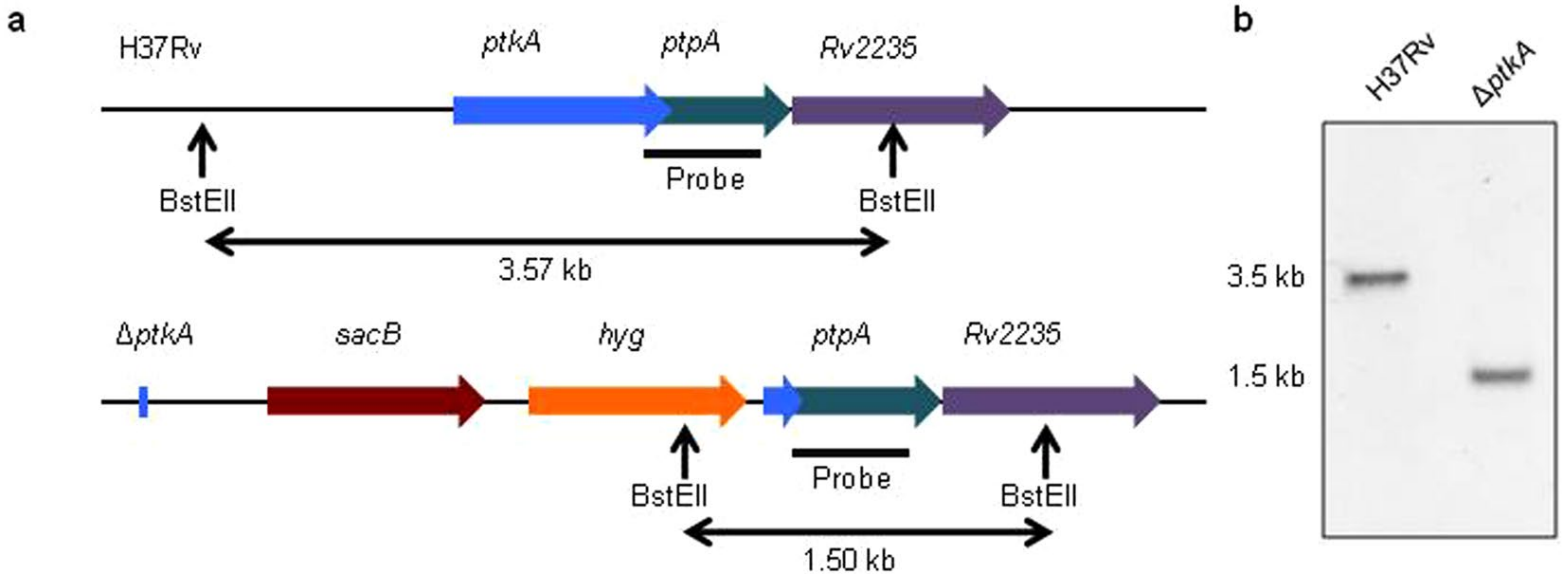
Construction and characterization of the ∆*ptkA* deletion mutant. (**a**) Map showing the *ptkA* genomic region and modification introduced during the ∆*ptkA* deletion mutant construction from the parental H37Rv strain. (**b**) Southern hybridization using a DIG-labeled probe hybridized to a 3.5 kb BstEII fragment for WT and a 1.5 kb fragment for the Δ*ptkA* mutant as predicted based on restriction map analysis.

To assess the role of PtkA in Mtb physiology, we constructed an Mtb mutant with a deletion of the gene encoding the PtkA tyrosine kinase by specialized phage transduction^24^. Genomic DNA isolated from the parental WT strain and the ∆*ptkA* mutant strain were subjected to Southern hybridization analysis to confirm that the gene encoding PtkA was replaced with the hygromycin resistance cassette as outlined in Fig. 1a and b. Whole genome sequencing confirmed successful replacement of the *ptkA* gene as designed without interruption of the rest of the genes within the *ptkA* operon^25^.

### Intracellular survival in THP-1 macrophages

**Figure 2:**
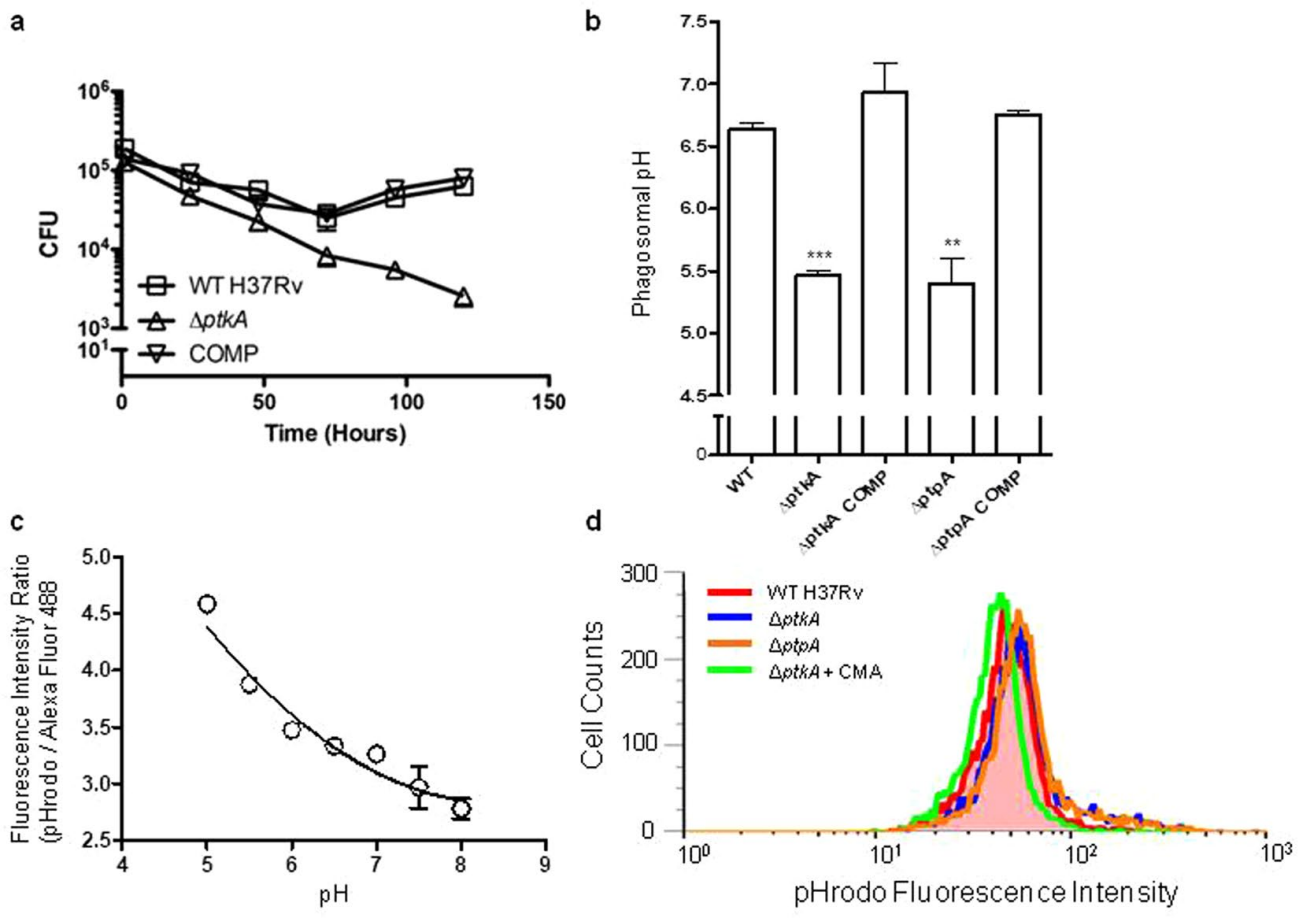
Intracellular survival in macrophages and inhibition of phagosome acidification. (**a**) Growth curve of WT, Δ*ptkA*, and complemented Mtb strains within THP-1 macrophage cell line showing an approximate 1-log reduction in Δ*ptkA* bacterial load compared to WT and complement at 6 days post-infection as measured by CFUs. (**b**) Phagosomal pH of THP-1 macrophages infected with WT, Δ*ptkA*, Δ*ptpA*, and corresponding complemented strains. Concanamycin (CMA)-treated macrophages served as a control. *p < 0.05; **p < 0.01; ***p < 0.001, significant difference compared with WT by Student’s t test, N = 3. (**c**) Calibration curve of phagosomal pH in THP-1 macrophages infected with Mtb dual-labeled with pHrodo and Alexa Fluor 488. The fluorescence intensity ratios of the fluorescence probes were calculated and plotted for each pH. (**d**) Overlaid FACS histograms of pHrodo fluorescence intensities of THP-1 macrophages infected with the indicated Mtb strains.

Since *ptkA* and *ptpA* are present in the same polycistronic operon, and PtpA is known to be essential for Mtb pathogenesis^15,20,26^ as well as being the phosphorylation substrate of PtkA^19,23^, we hypothesized that PtkA might also be required for Mtb intracellular growth within human macrophages. To test our hypothesis, we investigated the ability of the mutant strain to survive in the human THP-1 macrophage infection model. Indeed, the Δ*ptkA* strain was found to be attenuated within the macrophage, whereas the parental and the complemented strains were able to establish a stable infection after 3 days (Fig. 2a). At 6 days after infection, the mutant strain showed more than a log reduction in CFU compared to the WT strain. While the parental and complemented strains entered growth phase within the macrophage, the Δ*ptkA* strain was continuously cleared, establishing the importance of PtkA for Mtb growth within macrophages.

### Inhibition of the macrophage’s phagosomal acidification

In the case of the Δ*ptpA* mutant, impaired intracellular survival results from its inability to inhibit phagosome acidification^20^. To investigate whether this was the case with the Δ*ptkA* mutant, we examined phagosomal pH in THP-1 macrophages infected with the Δ*ptkA* mutant and compared it to its parental WT strain. We used fluorescence-activated cell sorting (FACS) to analyze the pH of Mtb-containing phagosomes. Parental and mutant strains were dually labeled with the pH-sensitive pHrodo fluorescent dye and the pH-insensitive Alexa Fluor 488. The Δ*ptpA* strain, shown earlier to be incapable of inhibiting phagosome acidification, was used as a control. As seen in Fig. 2b–d and Table 1, overlaid FACS histograms showed an increase in the mean pHrodo fluorescence intensity for phagosomes harboring the Δ*ptkA* strain (corresponding to pH 5.40), as compared to those containing the WT strain (corresponding to pH 6.64). This increase is similar to that observed for the Δ*ptpA* strain (corresponding to pH 5.46), indicating that the Δ*ptkA* strain failed to block phagosome acidification.

**Table 1:** Phagosomal pH of Mtb strains in THP-1 macrophages.

	pHrodo MFI	Alexa Fluor 488 MFI	Mean Fluorescence Ratio (pHrodo/AF488)	pH	Standard Error	*p* value
WT	44.80	13.77	3.25	6.64	0.08	NA
Δ*ptkA*	56.67	14.17	4.00	5.46	0.07	<0.0001
Δ*ptkA* COMP	45.45	14.50	3.13	6.93	0.33	NS
Δ*ptpA*	55.35	13.65	4.05	5.40	0.29	0.0046
Δ*ptpA* COMP	47.60	14.80	3.22	6.72	0.05	NS

COMP: Complement; AF488: Alexa Fluor 488; MFI: Mean Fluorescence Intensity; N/A: Not Applicable; NS: Not Significant.

### Increased cellular expression of *ptpA* and PtpA

**Figure 3:**
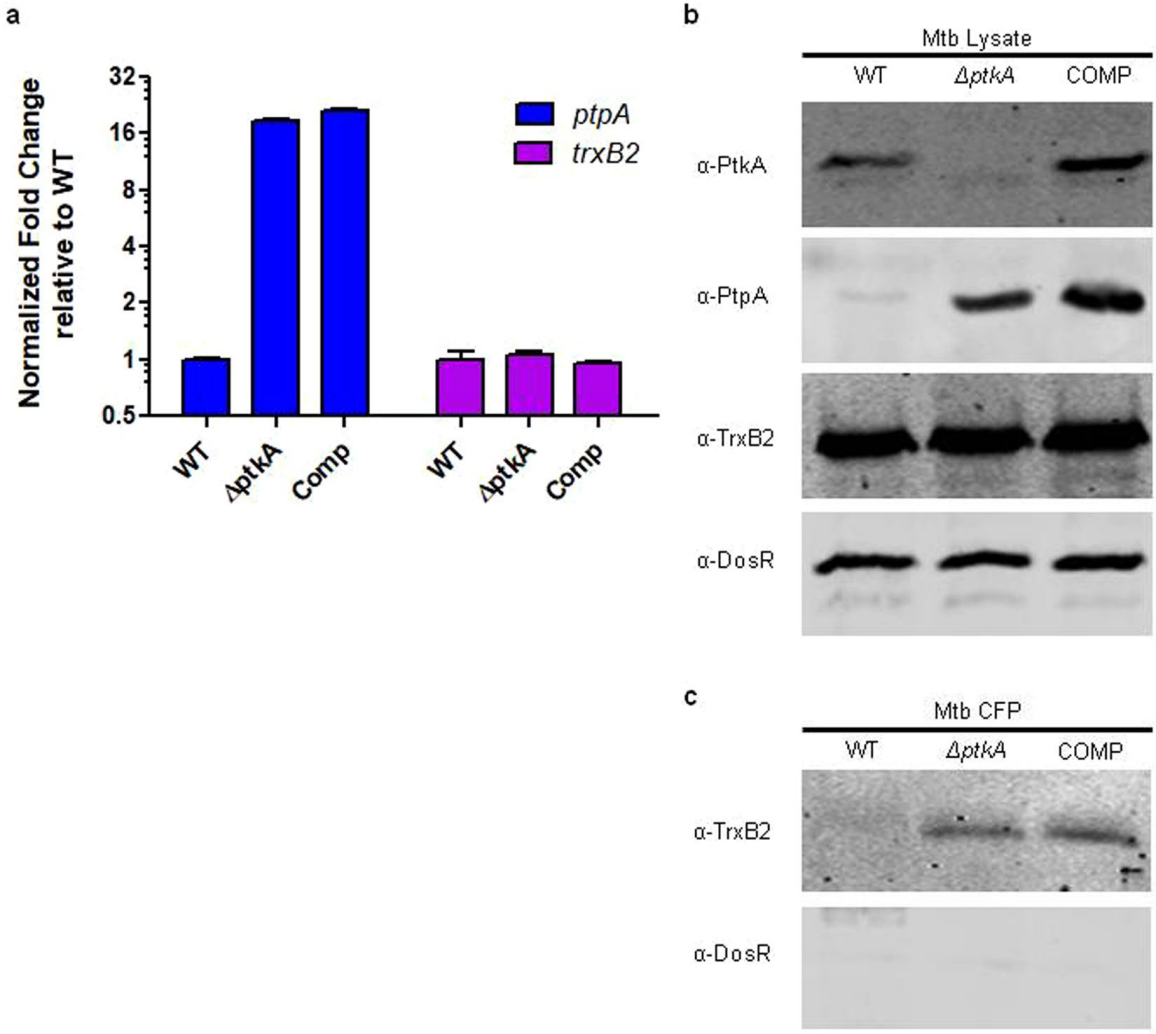
PtpA and TrxB2 expression profiles. (**a**) Quantitative real-time PCR analysis of *ptpA* and *trxB2*. cDNA expression levels were normalized to the house-keeping *sigA* gene. Values represent the fold increase compared to WT levels for each gene individually. Error bars show the standard deviation, N = 3. (**b**) Western analysis of PtkA (upper panel), PtpA (2^nd^ panel), and TrxB2 (3^rd^ panel) protein expression in WT, Δ*ptkA* and complement strains showing knockout of PtkA but increased levels of PtpA in the Δ*ptkA* mutant and complement. DosR (lower panel) was used as a loading control. (**c**) Western blot of TrxB2 from cellular lysate and culture filtrate fractions prepared from WT Mtb, Δ*ptkA* and complemented strains grown in Sauton’s media. The cytoplasmic DosR protein was used as a control for cell lysis in the culture filtrate fraction.

As the Δ*ptkA* and Δ*ptpA* strains demonstrated similar phenotypes in the macrophage infection, and because *ptkA* is the first gene in the *ptpA* operon, we checked whether *ptkA* deletion could have resulted in the loss of *ptpA* expression. Using quantitative real-time PCR (qPCR), we found that *ptpA* transcription was not disrupted but rather upregulated in the Δ*ptkA* mutant compared to WT Mtb (Fig. 3a). Western analysis confirmed increased levels of PtpA protein in the Δ*ptkA* mutant compared to WT (Fig. 3b, 2^nd^ panel). PtkA and the unrelated protein, DosR, were used as controls to confirm knockout of PtkA in the mutant and equal loading, respectively (Fig. 3b, 1^st^ and 4^th^ panels). Our iTRAQ experiment (below) also identified increased intracellular levels of both PtpA and Rv2235 (encoded in the same operon as *ptkA* and *ptpA*) in the Δ*ptkA* mutant. Complementation, however, did not restore WT levels of *ptpA* (Fig. 3a) or PtpA (Fig. 3b). Analysis of the genomic sequence of the Δ*ptkA* mutant suggests that upregulation of *ptpA* may be due to the introduction of a putative promoter sequence downstream of the hygromycin cassette^25^. Nevertheless, complementation did restore the macrophage infection phenotypes observed in the Δ*ptkA* mutant (Fig. 2). Therefore, while not ideal, the overexpression of *ptpA* in the mutant was a fortuitous, though accidental control, indicating that the similarity between the Δ*ptkA* and Δ*ptpA* strains is not due to loss of *ptpA* expression in the Δ*ptkA* mutant, but that PtkA is independently essential for Mtb pathogenesis.

### Resistance to oxidative stress in liquid cultures

**Figure 4:**
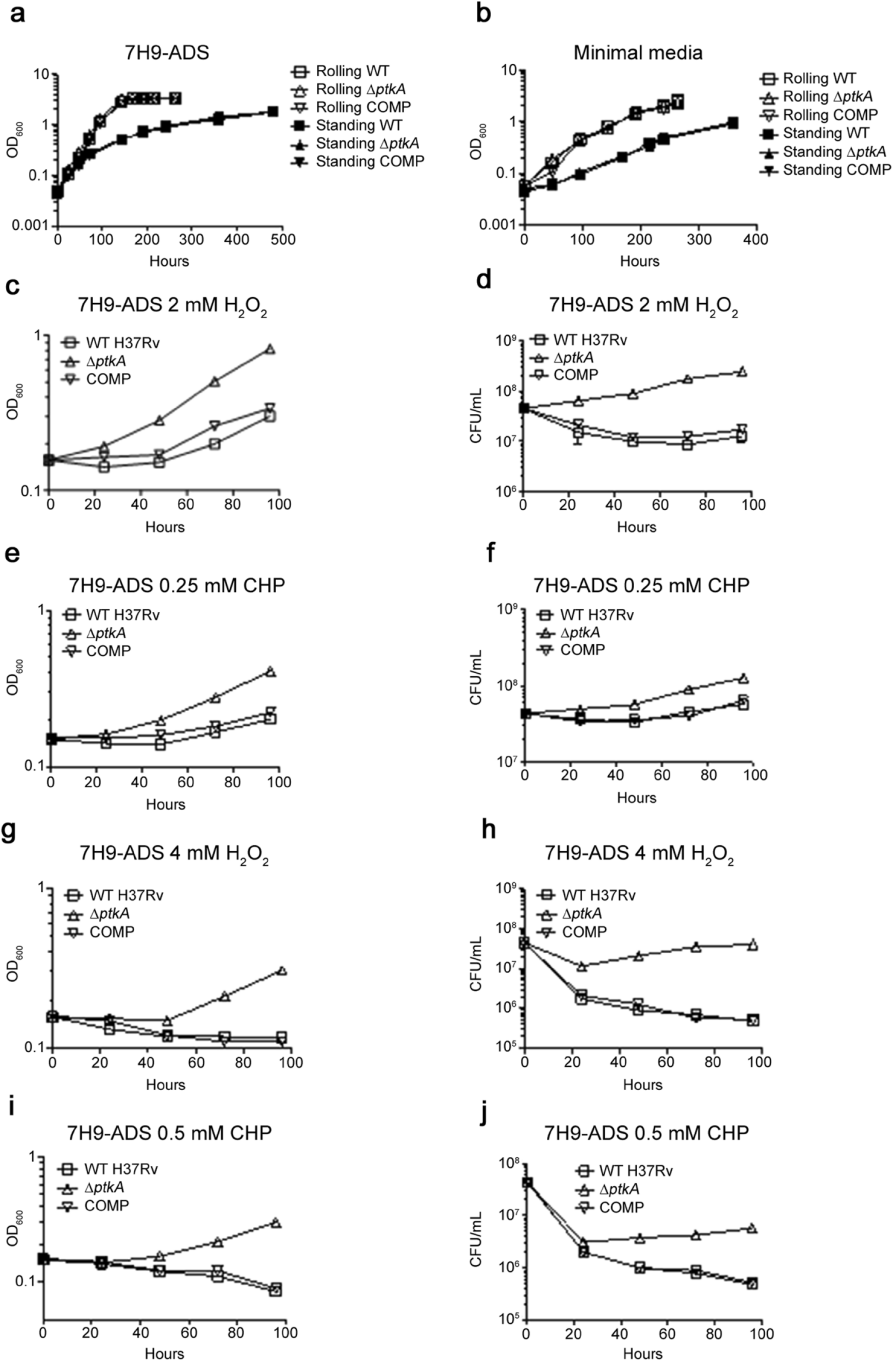
Increased resistance to oxidative stress. (**a**,**b**) *In vitro* growth of rolling and standing cultures of the parental WT, Δ*ptkA* and complemented (COMP) strains as measured by OD_600_ in 7H9 broth supplemented with 10% ADS and 0.05% Tween-80 (**a**) and minimal Sauton’s media supplemented with 0.05% Tween-80 (**b**). (**c**–**f**) Parental WT H37Rv, Δ*ptkA* mutant and COMP strains were grown in 7H9 supplemented with 10% ADS and 0.05% Tween-80 in the presence of 2 mM H_2_O_2_ (**c**,**d**), 0.25 mM cumene hydroperoxide (**e**,**f**), 4 mM H_2_O_2_ (**g**,**h**) or 0.50 mM cumene hydroperoxide (**i**,**j**). The OD_600_ (**c**,**e**,**g**,**i**) and CFUs (**d**,**f**,**h**,**j**) were measured every 24 hours for 5 days. CHP, cumene hydroperoxide.

To further characterize the Δ*ptkA* strain, we examined whether the deletion of the *ptkA* gene affected the growth of Mtb *in vitro*. The growth of the mutant with its parental and the complemented strains in various conditions was compared. No differences in growth rate were observed between the Δ*ptkA* mutant and its parental strain when grown as rolling or static cultures in 7H9-ADS or Sauton’s medium (Fig. 4a and b). As PtkA was shown to be phosphorylated by Ser/Thr protein kinases specifically under peroxide treatment^27^, and to mimic *in vivo* oxidative stress conditions, we tested the growth of the Δ*ptkA* mutant exposed to peroxides. Surprisingly, Δ*ptkA* growth, as measured by OD600 and colony forming units, was more resistant to sub-lethal and lethal concentrations of hydrogen peroxide (Fig. 4c,d,g and h) and cumene hydroperoxide (Fig. 4e,f,i, and j). Sensitivity to these oxidizing agents was suppressed by complementation of the Δ*ptkA* mutant with the WT gene, thus confirming that the mutated allele made the strain more resistant to oxidative stress *in vitro*. This *in vitro* phenotype appears to be in stark contrast to the mutant’s impaired intracellular survival.

### Proteomic analysis of the Δ*ptkA* mutant

**Figure 5:**
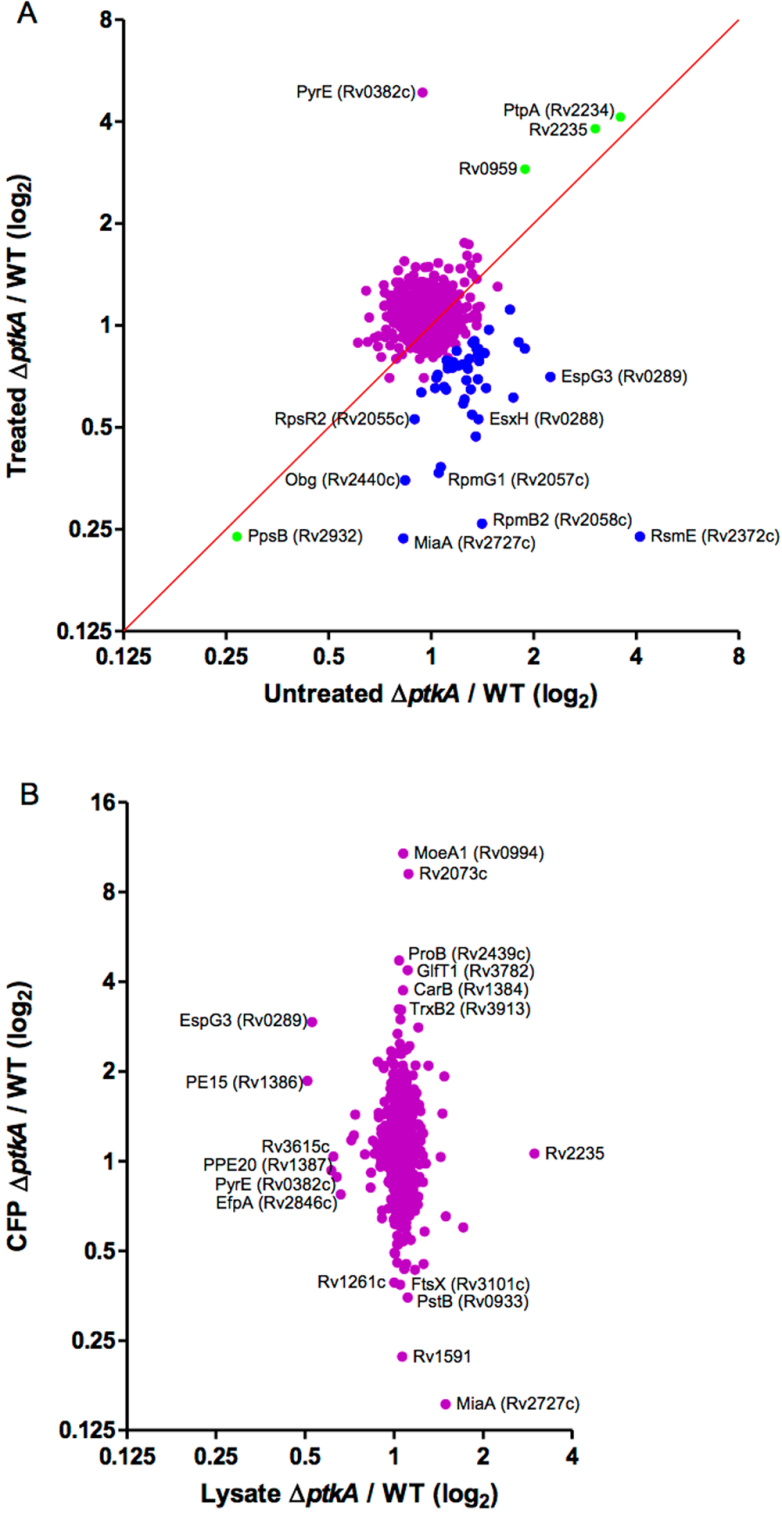
Graphical analysis of iTRAQ ratios. (**A**) Scatter plot comparison of untreated versus H_2_O_2_-treated ratios of Δ*ptkA*/WT grown in 7H9 media. Blue points indicate a 1.4-fold decrease in ptkA/WT ratios upon treatment with H_2_O_2_. Points that fall on the diagonal (green) indicate increased/decreased expression levels independent of H_2_O_2_ treatment. (**B**) Scatter plot of CFP versus cellular proteins of Δ*ptkA*/WT ratios grown in Sauton’s media. Selected proteins have been labeled.

To gain insight into potential regulation mediated by PtkA, we compared the protein expression profiles of Δ*ptkA* and WT Mtb with and without H_2_O_2_ treatment using the quantitative mass spectrometry proteomics approach, iTRAQ. We were able to identify and compare the levels of up to 1163 cellular proteins using a cutoff of 95% confidence in the identification of peptides. The ratios of individual protein levels ranged from 0.269 to 4.099 for untreated samples, and from 0.235 to 4.878 for H_2_O_2_-treated samples (Supplementary Data 1). Using an arbitrary cut-off of 1.5-fold change, 10 proteins were up-regulated and 8 proteins were down-regulated in untreated samples, while 11 were up-regulated and 21 down-regulated in treated samples. Graphical analysis of a plot of treated vs untreated Δ*ptkA*/WT ratios revealed that the largest subset of differentially expressed proteins mapped to the lower right quadrant, corresponding to a 1.4-fold decrease in Δ*ptkA* /WT ratios upon treatment with H_2_O_2_ (Fig. 5A, blue circles). This subgroup of 49 proteins was enriched for proteins related to the ESX type VII secretion systems and ribosome or ribosome-modifying proteins based on DAVID (Database for Annotation, Visualization and Integrated Discovery) gene ontology analysis. Further inspection also identified several proteins encoded in the zinc uptake regulator (Zur) regulon (Table 2). In addition to this subgroup, a lone protein involved in pyrimidine biosynthesis, PyrE, was found highly upregulated in the Δ*ptkA* mutant only upon treatment with H_2_O_2_ (Fig. 5A). Additionally, as noted above, the intracellular levels of PtpA and Rv2235 were highly upregulated in the Δ*ptkA* mutant independent of H_2_O_2_ treatment (Fig. 5A).

**Table 2:** Enriched protein groups based on differential protein levels in Δ*ptkA* in response to H_2_O_2_ treatment.

	Product/Function	Untreated *ΔptkA*/WT	Treated *ΔptkA*/WT	Ratio Fold Change	Regulon
EsxK (Rv1197)	CFP-10-like protein (ESX-5)^2^	1.308	0.648	2.02	
EsxL (Rv1198)	ESAT-6-like protein (ESX-5)^2^	1.122	0.764	1.47	
EsxN (Rv1793)	ESAT-6-like protein (ESX-5)^2^	1.232	0.766	1.61	
EsxO (Rv2346c)	ESAT-6-like protein (ESX-5)^2^	1.188	0.842	1.41	
EsxH (Rv0288)	ESAT-6-like protein (ESX-3)^2^	1.376	0.529	2.60	Zur
PPE3 (Rv0280)	PPE family secreted protein (ESX-3)^2^	1.119	0.747	1.50	Zur
EspG3 (Rv0289)	ESX-3 secretion-associated protein	2.24	0.705	3.18	Zur
Rv0106^1^	Hypothetical protein	1.036	0.701	1.48	Zur
RpmB2 (Rv2058c)	50S ribosomal protein L28	1.41	0.26	5.42	Zur
RpmG1 (Rv2057c)	50S ribosomal protein L33	1.053	0.367	2.87	Zur
RpsR2 (Rv2055c)	30S ribosomal protein S18	0.895	0.529	1.69	Zur
RsmI (Rv1003)	rRNA small subunit methyltransferase	1.357	0.857	1.58	
RsmE (Rv2372c)	rRNA small subunit methyltransferase	4.099	0.238	17.22	

^1^STRING analysis links *rv0106* to *rpmB2* based on putative homologs being neighbours and co-expressed other genomes. ^2^Secretory pathways are shown in brackets.

As the PtkA substrate, PtpA, is secreted, and Mtb secretes anti-oxidative defense enzymes, including a superoxide (SodA) and a catalase (KatG), we compared the culture filtrate proteins (CFPs) of Δ*ptkA* and WT. iTRAQ analysis of CFPs revealed 877 proteins identified with 95% confidence. Of these, 29 proteins were found to be increased by at least 2-fold in the Δ*ptkA* mutant, while 12 proteins decreased, with levels ranging from 0.153 to 10.784 (Supplementary Data 1). CFP levels were plotted against the corresponding cellular protein levels of Mtb grown in Sauton’s media and shown in Fig. 5B. Of note, the thioredoxin reductase TrxB2 was found to be 3.2-fold higher in protein abundance in the culture filtrate of the Δ*ptkA* strain. Since the thioredoxin system participates in the detoxification of ROI^13^ and therefore may account for the enhanced resistance to oxidative stress observed in the Δ*ptkA* mutant, we decided to further investigate the link between PtkA and TrxB2.

### TrxB2 expression and phosphorylation

The increased secretion level of TrxB2 was confirmed by Western blot analysis of CFPs (Fig. 3c, upper panel). The absence of Mtb autolysis during culturing was confirmed by Western blot analysis of the harvested CFPs with antibodies against the cytosolic DosR protein (Fig. 3c, lower panel). However, both expression of the *trxB2* gene (Fig. 3a) as well as the intracellular protein levels of TrxB2 (Fig. 3b) revealed no difference in the Δ*ptkA* mutant compared to WT and complement strains.

**Figure 6:**
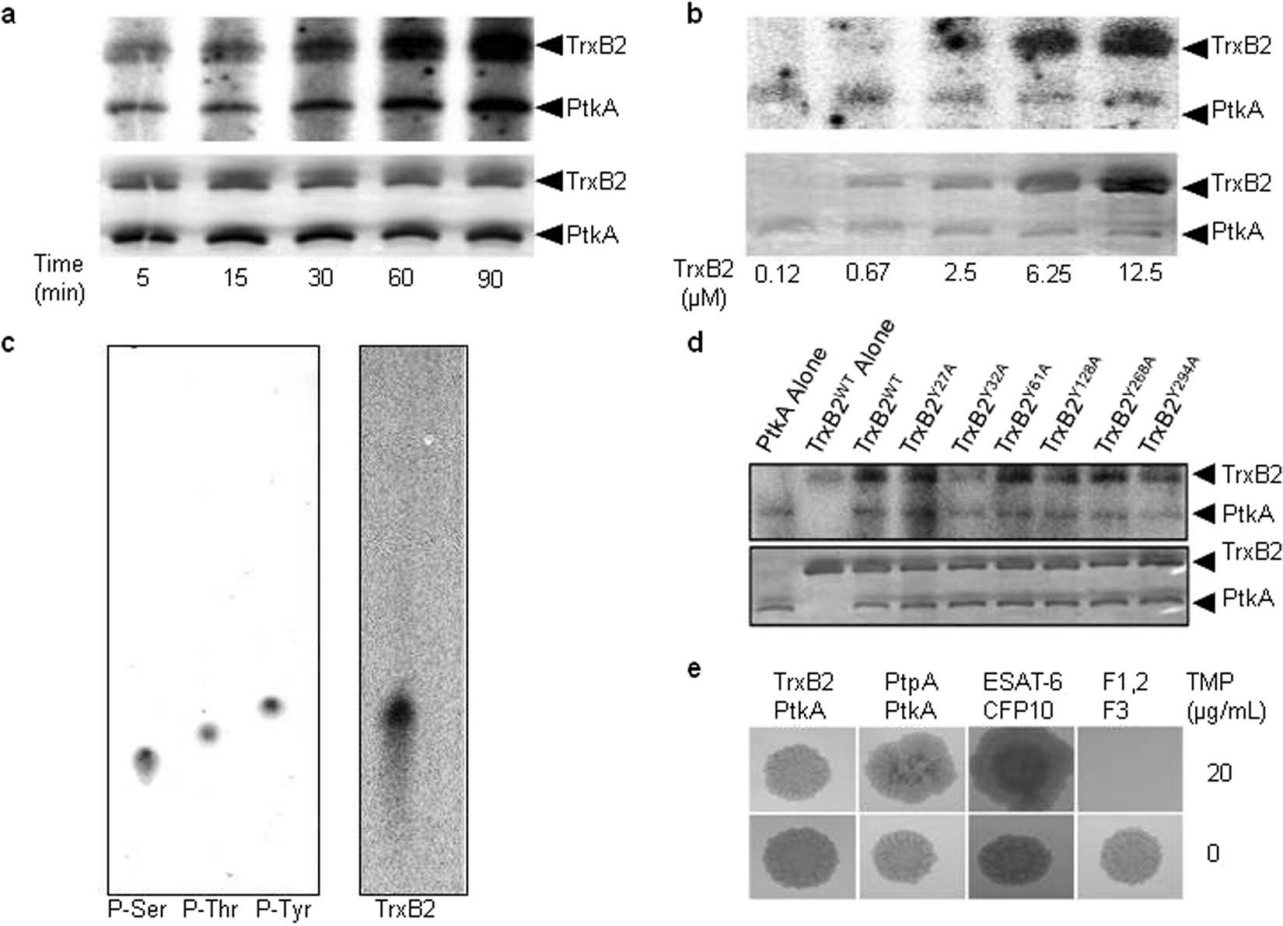
TrxB2 tyrosine phosphorylation by PtkA. (**a**,**b**) *In vitro* kinase assays demonstrating time-dependent (**a**) and dose-dependent (**b**) phosphorylation of TrxB2 by PtkA using [γ-^32^P]ATP. Upper panel, phosphorimage; lower, silver-stained SDS-PAGE. (**c**) Phosphoamino acid analysis of TrxB2 phosphorylated by PtkA *in vitro*. Retention factor (Rf): Phospho-serine (P-Ser), 0.255; Phospho-threonine (P-Thr), 0.300; Phospho-tyrosine (P-Tyr), 0.345; TrxB2, 0.339. (**d**) *In vitro* kinase assays with [γ-^32^P]ATP of PtkA-phosphorylated TrxB2 recombinant proteins with single amino acid Tyr-Ala point mutations generated by site-directed mutagenesis of *trxB2*. (**e**) Mycobacterial protein fragment complementation assay demonstrating interaction of the indicated recombinant proteins fused to the F1,2 or F3 domains of mDHFR. Reassembly of the mDHFR domains enabled growth of *M. smegmatis* in the presence of 20 μg/mL trimethoprim (TMP). Identical spots on control plates without trimethoprim revealed growth of all strains (lower panel). Positive Control, Mtb ESAT-6 and CFP10 fused to F1,2 and F3, respectively; Negative Control, mDHFR fragments alone.

Given that PtpA is secreted and is a substrate of PtkA^19^, we tested the possibility that TrxB2, being secreted and dysregulated in the Δ*ptkA* mutant, may also serve as a substrate for PtkA. Indeed, *in vitro* kinase assays demonstrated that PtkA phosphorylated TrxB2 in a dose- and time-dependent manner (Fig. 6a,b). Phosphoamino acid analysis of the phosphorylated TrxB2 protein further revealed that PtkA phosphorylated TrxB2 on tyrosine, consistent with PtkA’s tyrosine kinase activity (Fig. 6c). We further performed site-directed mutagenesis to examine which Tyr residue in TrxB2 was phosphorylated. Mutation of Tyr^32^ significantly reduced phosphorylation of TrxB2, indicating that Tyr^32^ is targeted by PtkA for phorphorylation (Fig. 6d).

To further verify the kinase-substrate interaction in a cellular model, PtkA and TrxB2 were subjected to the mycobacterial protein fragment complementation assay^28^. Co-expression of TrxB2-F3 with PtkA-F1,2 resulted in reconstitution of mDHFR activity, as evidenced by growth of *M. smegmatis* on media supplemented with trimethoprim, confirming PtkA interaction with TrxB2 (Fig. 6e). The interaction between PtkA and TrxB2 was found to be similar to that of PtkA and PtpA, based on comparable *M. smegamatis* growth in the presence of trimethoprim.

## Discussion

Tyr phosphorylation in mycobacteria has long been a subject of interest^29^. Our results^19^, along with studies from others^30,31^, have demonstrated Tyr kinase activity in Mtb. However, the physiological function of Tyr phosphorylation in Mtb has not been explored. We have previously shown that PtkA represents a novel prokaryotic protein Tyr kinase^19,32^. PtkA was also proposed to play a key role in Mtb pathogenesis^21,32^ based on its substrate, PtpA, being essential for preventing phagosome acidification and maturation^15,20^. Here, we examined the role of PtkA in Mtb pathophysiology and pathogenesis by generating a mutant Mtb strain with a deletion of the *ptkA* gene. The Δ*ptkA* mutant was impaired in intracellular survival within the THP-1 macrophage infection model and is consistent with the mutant’s failure to inhibit phagosomal acidification. These results match the phenotype of the Δ*ptpA* deletion mutant^15,20^ suggesting that PtkA inhibition of phagosomal acidification may be mediated via PtpA function, either through PtkA phosphorylation of PtpA, which enhances PtpA phosphatase activity^23^, and/or via PtkA-dependent PtpA secretion. Further experiments are needed to test this hypothesis.

While *ptkA* was needed for intracellular survival, the lack of *ptkA* enhanced the mutant stain’s resistance to oxidative stress *in vitro*. Proteomic analyses identified the thioredoxin reductase, TrxB2, to have increased secretion in the Δ*ptkA* mutant. While the exact mechanism of this upregulation was not identified and may be a result of increased expression of PtpA and/or Rv2235 as complementation of the *ptkA* mutant did not restore WT levels, we found that PtkA is able to bind and phosphorylate TrxB2 on tyrosine. Additionally, we demonstrated that TrxB2 is secreted, which has not been reported previously to our knowledge. Secretion of TrxB2 is consistent with the finding that TrxC, the thioredoxin substrate of TrxB2, is secreted^33–36^, as we also observed (Supplementary Data 1).

While it is tempting to speculate that increased secretion of TrxB2 is responsible for the mutant’s increased resistance to oxidative stress, the fact that TrxB2 secretion was also upregulated in the complement, while complementation restored the WT sensitivity to oxidative stress suggests an alternative mechanism is at play. Furthermore, the relatively modest 3.2-fold increased secretion of TrxB2, is unlikely to be able to result in the up-to 2-log enhanced growth/survival of the Δ*ptkA* mutant in lethal concentrations of oxidative stress (Fig. 4). Other variations in protein levels identified in the iTRAQ experiment, such as the decrease in ribosomal proteins and rRNA methyltransferases observed in the Δ*ptkA* mutant (Table 2), may lead to a slower metabolism and thus increased resistance to oxidative stress. Alternatively, in *S. aureus*, the methylation status of rRNA has been shown to affect resistance to oxidative stress^37^. However, we did not establish a direct role for PtkA in this downregulation which may be an indirect downstream effect of the oxidative stress.

A second cluster of enriched proteins that were identified to be dysregulated in the Δ*ptkA* mutant when exposed to H_2_O_2_ belong to the ESX type 7 secretion systems (Table 2). Interestingly, each of these proteins are predicted to be secreted by either the ESX-3 or ESX-5 secretion systems^38^. Consistent with this finding, the structural components of the ESX secretion systems that were identified in our iTRAQ analysis showed little to no difference in protein levels between WT and mutant (Supplementary Data 1). Given that the two identified substrates of PtkA, PtpA^19^ and TrxB2 (this study), are secreted, and that the dysregulated ESX proteins are secretion proteins, suggests that PtkA may play a role in secretion.

The ESX-5 secretion system has been described to be involved in nutrient uptake^39^, while the ESX-3 system is necessary for iron and zinc homeostasis^40^. Interestingly both the identified ribosomal proteins and the ESX-3 proteins are encoded in the zur regulon which responds to zinc levels^41^. Disruption of metalloregulatory proteins can result in a change to intrabacterial redox state^42^ and may contribute to the mutant’s resistance to oxidative stress, as over-exposure to metal ions could render them highly toxic through interference with the functioning of macromolecules and the generation of free radicals^43^.

While the Δ*ptkA* mutant demonstrated enhanced resistance to oxidative stress, this resistance was not sufficient to compensate for or overcome the mutant’s inability to block phagosomal acidification in the macrophage infection model, and the failure to grow and survive within the macrophage. Additionally, Mehra *et al*.^44^ discovered that the EsxG-EsxH complex, secreted by the ESX-3 system, interacts and interferes with the endosomal sorting complex required for transport (ESCRT), demonstrating a direct role for ESX-3 in the arrest of phagosome maturation. In this regard, the downregulation of EsxH identified in the Δ*ptkA* mutant could also potentially lead to the failure in blocking phagosome-lysosome fusion during macrophage infection and explain the impaired intracellular survival phenotype observed for the Δ*ptkA* strain. Further experiments are needed to test this hypothesis.

To conclude, our studies established a central role for PtkA in macrophage infection by Mtb and correlated the enzyme activity to phosphorylation and secretion of TrxB2 and PtpA.

## Methods

### Strains, media and conditions

Mtb H37Rv and their derivative strains were grown on Middlebrook 7H10 agar (BD Diagnostic Systems, Mississauga, ON, Canada) supplemented with 10% (v/v) OADC (oleic acid, albumin, dextrose and catalase solution). For liquid cultures, Mtb strains were grown in Middlebrook 7H9 broth (BD Diagnostic Systems) supplemented with 10% (v/v) OADC or ADS (albumin, dextrose and NaCl solution) and 0.05% (v/v) Tween-80 (Sigma-Aldrich) or Sauton’s media supplemented with 0.05% (v/v) Tween-80 at 37 °C standing or rolling in incubator. *Escherichia coli* strains were cultured in Luria-Bertani (LB) broth. The concentrations of antibiotics used were 75 μg/ml hygromycin and 20 μg/ml kanamycin for mycobacterial strains, and 150 μg/ml hygromycin and 40 μg/ml kanamycin for *E. coli*.

### Construction of *ΔptkA* deletion mutant

Construction of the Δ*ptkA* deletion mutant was performed according to the specialized transduction with conditionally replicating mycobacteriophage method described previously^24^. Briefly, 1000-bp of the upstream and downstream flanking regions of the *ptkA* gene were PCR-amplified from genomic DNA of Mtb H37Rv. Subsequently, the upstream and downstream flanks were digested with the indicated restriction enzymes, and ligated with Van91I-digested p0004-SacB vector containing the γδ*res*-sacB-hyg-γδ*res* cassette comprising of the *sacB* gene and hygromycin resistance gene flanked by *res*-sites of the γδ-resolvase. The resulting knockout plasmids were then linearized with PacI and cloned and packaged into the temperature-sensitive phage phAE159, as previously described^24^, yielding knockout phages which were propagated in *M. smegmatis* at 30 °C. The mycobacteriophages harboring the knockout constructs were harvested and used for allelic exchange with specialized transduction as reported previously. Mtb H37Rv cultures were grown to an OD_600_ of 0.8–1.0, washed with 7H9-glycerol-ADS and resuspended in buffer before mixing with the mycobacteriophages at a multiplicity of infection of 10:1 (mycobacteriophage: mycobacteria), and the mixture was incubated at 37 °C overnight. The cells were pelleted and resuspended in 7H9-glycerol-ADS and plated in three 7H10-glycerol-OADC plates with 50 μg/mL of hygromycin and incubated at 37 °C to select for gene deletion and replacement by the γδres-sacB-hyg-γδres cassette. Putative Δ*ptkA* clones were screened by PCR using two pairs of oligonucleotides targeting the hygromycin gene and region flanking the hygromycin cassette. Based on the PCR analysis, two isolates were chosen for Southern hybridization analysis. Genomic DNAs isolated from the parental WT and the two isolates of the Δ*ptkA* strain were subjected to Digoxigenin (DIG)-based Southern hybridization analysis to confirm *ptkA* deletion.

### Complementation of *ΔptkA* strain

For complementation of Δ*ptkA*, the DNA sequence encoding WT PtkA under the control of the native promoter was cloned into the pKP186 integrative vector. The Δ*ptkA* strain was co-transformed with the resulting plasmid and pBS*int*, a non-replicating plasmid that provides integrase in *trans* but is subsequently lost from the cells, thereby reducing the chances of integrase-mediated excision of the complementing DNA. Transformants were selected on 7H10 agar with 10% OADC and 50 μg/mL kanamycin.

### Hydrogen peroxide sensitivity assay

Mtb cultures at OD_600_ = 0.15 in 7H9 media supplemented with 0.05% (v/v) Tween-80 and ADS or Sauton’s media supplemented with 0.05% (v/v) Tween-80 were exposed to a single dose of H_2_O_2_ (0 mM–4 mM) *in vitro*. Bacterial growth (OD_600_) and colony forming unit (CFU) were monitored every 24 hours after addition of stress. Three independent experiments were conducted for each H_2_O_2_ concentration.

### Infection of THP-1

Infection of THP-1 macrophages was performed using human serum-opsonized Mtb at a multiplicity of infection (MOI) of 10:1 or 1:1 (Bacteria: Macrophage). For Mtb intracellular survival studies, infected macrophages were harvested at defined time points, lysed with 0.025% SDS, serial diluted, and plated on 7H10 agar medium supplemented with OADC and appropriate antibiotics. The plates were incubated at 37 °C for 2 weeks until colonies could be counted.

### Measurement of phagosomal pH in THP-1 macrophages infected with Mtb

Phagosomal pH measurement was performed as described previously^20^. Briefly, THP-1 macrophages were seeded on 6 well plates (1.0 × 10^6^) with 40 ng/ml PMA. Mtb strains were first labeled with 20 M pHrodo succinimidyl ester (SE) (Invitrogen) at 37 °C for one hour. The bacteria were washed with 7H9 supplemented with 0.05% Tween-80 three times before being labeled with 25 g/mL Alexa Fluor 488 carboxylic acid SE (Invitrogen). The bacteria were then washed and opsonized with human serum. The THP-1 macrophages were subsequently infected with the labeled Mtb at a MOI of 10:1 for 2 hours at 37 °C with 5% CO_2_. Non-internalized bacteria were washed away, and the infection was carried out for another 2 hours at 37 °C with 5% CO_2_. For pH calibration, the infected cells were incubated in 10 mM phosphate-citrate buffer with pre-determined pH (5.0–8.0) (Fig. S1A and S1B). The cells were then washed, scraped off the plate and fixed with 2.5% paraformaldehyde. The fixed cells were then analyzed with FACS on FACSCalibur (BD Bioscience) and the FlowJo 8.7 software. Color compensation was performed to prevent signal overlapping. Mean fluorescence intensities of pHrodo and Alexa Fluor 488 were used to calculate phagosomal pH.

### Protein preparation for proteomics analysis

Mtb H37Rv and their derivative strains were grown in Middlebrook 7H9 broth (BD Diagnostic Systems) supplemented with 10% (v/v) OADC or ADS and 0.05% (v/v) Tween-80 (Sigma-Aldrich) to an OD_600_ = 0.6. The cultures were washed with 7H9 or Sauton’s media and inoculated into fresh 7H9-ADS or Sauton’s media supplemented with 0.05% (v/v) Tween-80. Sauton’s media was used when culture filtrate proteins were analyzed. 2 mM H_2_O_2_ was added when appropriate, and the cultures were grown at 37 °C rolling in incubator until OD_600_ = 1.0. The culture filtrate proteins were harvested by centrifuging the Mtb cultures to obtain the spent culture supernatant. The supernatant was filtered through a 0.22 μm syringe filter unit (Corning) before removal from BSL-3 laboratory. The filtered supernatant was then concentrated with a stirred cell concentrator (EMD Millipore) fitted with YM-3 membrane (3 kDa-cutoff). Cytosolic fractions were extracted by lysing Mtb through bead-beating. The lysate was centrifuged, and the supernatant was filtered through a 0.22 μm syringe filter unit before removal from BSL-3 laboratory.

### iTRAQ (isobaric tags for relative and absolute quantitation)

Protein concentrations were determined using a bicinchonic acid protein assay (Sigma). Samples (85 µg of each) were precipitated overnight in acetone at 4 °C followed by resolubilization in 0.5 M triethylammoniumbicarbonate, 0.2% SDS. Proteins were reduced with tris-(2-carboxyethyl)phosphine and alkylated with methyl methanethiosulfonate Proteins were then “in-solution” digested with trypsin (Promega) and labeled with the appropriate iTRAQ label. iTRAQ labeled peptides were then combined and separated by strong cation exchange HPLC. HPLC fractions containing peptides were then reduced in volume by speed-vac and analyzed by LC-MS/MS. The length of the reverse gradient used was 2 hours per HPLC fraction. Samples were analyzed by reversed phase nanoflow (300 nL/min) HPLC with nano-electrospray ionization using a LTQ-Orbitrap mass spectrometer (LTQ-Orbitrap Velos, Thermo-Fisher) operated in positive ion mode. All data was analyzed using Proteome Discoverer v1.3 (Thermo-Fisher) and MASCOT v2.3 (Matrix Science). Raw data files were searched against the Uniprot-SwissProt database with “All Species” filter and *Mycobacterium tuberculosis* complex species only.

### RNA extraction and real-time quantitative PCR

WT Mtb and Δ*ptkA* Mtb strains were grown in rolling cultures at 37 °C in Sauton’s medium. Bacteria were harvested at OD~1.0 and RNAs were extracted using FastRNA Pro Blue kit (MP Biomedicals). Cells were lysed in the presence of glass beads in a FastPrep ribolyzer (MP Biomedicals) according to manufacturer’s instructions. Post lysis, RNA extraction was performed with chloroform, and after ethanol precipitation, RNA was dissolved in nuclease-free water. Purified RNA was treated with RNase-free *Turbo* DNase (Ambion) according to the supplier’s instructions in order to digest contaminating DNA and the Illustra RNAspin minikit (GE Healthcare) was used for the subsequent cleanup procedures. Removal of DNA was confirmed by performing PCR using an aliquot of the DNase-treated RNA as a template. Reverse transcription reactions were carried out in 20-μl volumes containing 2 μg RNA, random primers, and the buffer and enzyme components of the EasyScript cDNA Synthesis kit (ABM) according to the supplied protocol. Real-time PCR analysis was carried out on the Step One Real Time PCR system (Life Technologies). Each qPCR reaction contained 2X FastStart SYBR green master mix (Roche), 15ng cDNA and 0.3 μM of each primer. Control reactions without reverse transcriptase were also included with each run to confirm the absence of genomic DNA contamination. Expression level was normalized to the constitutively expressed *sigA* gene.

### Western blot analysis

Western blot analyses were performed according to standard protocols. Briefly, 26–30 µg of cellular extracts and CFP of the indicated Mtb strains were resolved by SDS-PAGE and transferred onto a nitrocellulose membrane (Bio-Rad). The blots were blocked with 2% milk proteins in TBS-T and probed with affinity purified rabbit polyclonal (YenZym Antibodies, LLC) anti-PtkA (1:50000), anti-PtpA (1:500), anti-TrxB2 (1:50000), or anti-DosR (1:2500) and incubated overnight at 4 °C. For detection, Alexa Fluor 680 goat anti-rabbit (ThermoFisher) antibody was used as the secondary detection reagent (1:10,000), and detection was done using an Odyssey Infrared CLx Imager (LI-COR Biosciences). Brightness and contrast were adjusted and applied to entire images using Adobe Photoshop CS5.1. Full-length images are shown in Supplementary Fig. 1.

### *In vitro* kinase assay

Kinase assays were carried out using [γ-^32^P]ATP as the phosphate donor according to published protocols^45^ with some modifications. The *in vitro* kinase reactions contained 20 mM Tris-HCl, pH = 7.5, 1 mM MnCl_2_, 0.1 mM MgCl_2_ and 1 mM DTT. Proteins (1.3–1.4 µM of PtkA and 1.2 µM of TrxB2 for the time-dependent assay and 3.4 µM of TrxB2 for the single amino acid point mutation kinase assay) were separated by SDS/PAGE (12%) supplemented with 8 M urea, and silver-stained. The ^32^P-radioactively labeled protein bands were detected using a PhosphorImager SI (Molecular Dynamics). Brightness and contrast were adjusted and applied to entire autoradiography images using Adobe Photoshop CS5.1. Full-length images are shown in Supplementary Fig. 2. Densitometric analysis of TrxB2 phosphorylation in Fig. 6d using Adobe Photophop CS5.1 is shown in Supplementary Fig. 3.

### Phosphoamino acid analysis

Recombinant PtkA was incubated with [γ-^32^P]ATP in the kinase buffer described above. The sample was hydrolyzed with 6 N HCl at 110 °C for 1 hour and separated on a cellulose TLC plate by ascending chromatography^45^. For TrxB2 phosphorylation by PtkA, the reaction was stopped with sample buffer, loaded into SDS-PAGE (12%) containing 8 M urea, and subjected to electrophoresis. The gel was electroblotted on to an Immobilon PVDF membrane^46^. Phosphorylated proteins bound to the membrane were detected by autoradiography using a PhosphorImager apparatus. The ^32^P-labelled protein band corresponding to the migration of TrxB2 was excised from the membrane and hydrolyzed following the same procedure as described above. After hydrolysis, samples were separated on ascending TLC chromatography. After migration, radioactive amino acids were detected by autoradiography. Brightness and contrast were adjusted and applied to entire autoradiography images using Adobe Photoshop CS5.1. Phosphoserine, phosphothreonine and phosphotyrosine standards (Sigma) were separated on cellulose TLC plate in parallel and visualized by staining with ninhydrin.

### Mycobacterial protein fragment complementation assay

The mycobacterial protein fragment complementation assay was performed as described^28^. The genes of interest were PCR-amplified and cloned into pUAB100 (expressing mDHFR fragment F1,2) or pUAB200 (expressing mDHFR fragment F3). *M. smegmatis* was co-transformed with both plasmids, and the co-transformants were selected on 7H11 agar plates with 25 μg/mL kanamycin and 50 μg/mL hygromycin and tested for growth over 3–4 days on 7H11 kanamycin/hygromycin plates supplemented with 20 μg/mL trimethoprim.

### Data availability

All relevant data are within the paper and its Supporting Information file.

## Electronic supplementary material

Supplementary Information

Supplementary Dataset

## References

[1] Dye C, Williams BG (2010). The population dynamics and control of tuberculosis. Science.

[2] Shenoi S, Heysell S, Moll A, Friedland G (2009). Multidrug-resistant and extensively drug-resistant tuberculosis: consequences for the global HIV community. Curr Opin Infect Dis.

[3] Pryor PR, Luzio JP (2009). Delivery of endocytosed membrane proteins to the lysosome. Biochimica et biophysica acta.

[4] Tjelle, T. E., Lovdal, T. & Berg, T. Phagosome dynamics and function. *Bioessays* **22**, 255–263, doi:10.1002/(SICI)1521-1878 (2000). 10.1002/(SICI)1521-1878(200003)22:3<255::AID-BIES7>3.0.CO;2-R 10684585

[5] Sturgill-Koszycki S (1994). Lack of acidification in Mycobacterium phagosomes produced by exclusion of the vesicular proton-ATPase. Science.

[6] Poirier, V. & Av-Gay, Y. Intracellular Growth of Bacterial Pathogens: The Role of Secreted Effector Proteins in the Control of Phagocytosed Microorganisms. *Microbiol Spectr* **3**, 10.1128/microbiolspec.VMBF-0003-2014 (2015). 10.1128/microbiolspec.VMBF-0003-2014 27337278

[7] Hmama Z, Pena-Diaz S, Joseph S, Av-Gay Y (2015). Immunoevasion and immunosuppression of the macrophage by Mycobacterium tuberculosis. Immunol Rev.

[8] Poirier V, Av-Gay Y (2012). Mycobacterium tuberculosis modulators of the macrophage’s cellular events. Microbes Infect.

[9] Fratti RA, Chua J, Vergne I, Deretic V (2003). Mycobacterium tuberculosis glycosylated phosphatidylinositol causes phagosome maturation arrest. Proc Natl Acad Sci USA.

[10] Vergne I (2005). Mechanism of phagolysosome biogenesis block by viable Mycobacterium tuberculosis. Proc Natl Acad Sci USA.

[11] Manca C, Paul S, Barry CE, Freedman VH, Kaplan G (1999). Mycobacterium tuberculosis catalase and peroxidase activities and resistance to oxidative killing in human monocytes *in vitro*. Infect Immun.

[12] Bryk R, Lima CD, Erdjument-Bromage H, Tempst P, Nathan C (2002). Metabolic enzymes of mycobacteria linked to antioxidant defense by a thioredoxin-like protein. Science.

[13] Jaeger T (2004). Multiple thioredoxin-mediated routes to detoxify hydroperoxides in Mycobacterium tuberculosis. Arch Biochem Biophys.

[14] Rawat M (2002). Mycothiol-deficient Mycobacterium smegmatis mutants are hypersensitive to alkylating agents, free radicals, and antibiotics. Antimicrob Agents Chemother.

[15] Bach H, Papavinasasundaram KG, Wong D, Hmama Z, Av-Gay Y (2008). Mycobacterium tuberculosis virulence is mediated by PtpA dephosphorylation of human vacuolar protein sorting 33B. Cell Host Microbe.

[16] Hestvik AL, Hmama Z, Av-Gay Y (2003). Kinome analysis of host response to mycobacterial infection: a novel technique in proteomics. Infect Immun.

[17] Hestvik AL, Hmama Z, Av-Gay Y (2005). Mycobacterial manipulation of the host cell. FEMS Microbiol Rev.

[18] Av-Gay, Y. & Everett, M. The eukaryotic-like Ser/Thr protein kinases of Mycobacterium tuberculosis. *Trends Microbiol* **8**, 238–244, doi:S0966-842X(00)01734-0 [pii] (2000). 10.1016/s0966-842x(00)01734-0 10785641

[19] Bach H, Wong D, Av-Gay Y (2009). Mycobacterium tuberculosis PtkA is a novel protein tyrosine kinase whose substrate is PtpA. Biochem J.

[20] Wong D, Bach H, Sun J, Hmama Z, Av-Gay Y (2011). Mycobacterium tuberculosis protein tyrosine phosphatase (PtpA) excludes host vacuolar-H+ -ATPase to inhibit phagosome acidification. Proc Natl Acad Sci USA.

[21] Wong D, Chao JD, Av-Gay Y (2013). Mycobacterium tuberculosis-secreted phosphatases: from pathogenesis to targets for TB drug development. Trends Microbiol.

[22] Chao J (2010). Protein kinase and phosphatase signaling in Mycobacterium tuberculosis physiology and pathogenesis. Biochimica et biophysica acta.

[23] Zhou P, Li W, Wong D, Xie J, Av-Gay Y (2015). Phosphorylation control of protein tyrosine phosphatase A activity in Mycobacterium tuberculosis. FEBS Lett.

[24] Bardarov S (2002). Specialized transduction: an efficient method for generating marked and unmarked targeted gene disruptions in Mycobacterium tuberculosis, M. bovis BCG and M. smegmatis. Microbiology.

[25] Tsui, C. K. M. *et al*. Genome Sequences of the Mycobacterium tuberculosis H37Rv-ptkA Deletion Mutant and Its Parental Strain. *Genome Announc* **5**, 10.1128/genomeA.01156-17 (2017). 10.1128/genomeA.01156-17 PMC5668532 29097456

[26] Cowley, S. C., Babakaiff, R. & Av-Gay, Y. Expression and localization of the Mycobacterium tuberculosis protein tyrosine phosphatase PtpA. *Res Microbiol* **15**3, 233–241, doi:S0923-2508(02)01309-8 (2002). 10.1016/s0923-2508(02)01309-8 12066895

[27] Prisic S (2010). Extensive phosphorylation with overlapping specificity by Mycobacterium tuberculosis serine/threonine protein kinases. Proc Natl Acad Sci USA.

[28] Singh A, Mai D, Kumar A, Steyn AJ (2006). Dissecting virulence pathways of Mycobacterium tuberculosis through protein-protein association. Proc Natl Acad Sci USA.

[29] Chow K, Ng D, Stokes R, Johnson P (1994). Protein tyrosine phosphorylation in Mycobacterium tuberculosis. FEMS microbiology letters.

[30] Stehle T (2012). The apo-structure of the low molecular weight protein-tyrosine phosphatase A (MptpA) from Mycobacterium tuberculosis allows for better target-specific drug development. J Biol Chem.

[31] Kusebauch U (2014). Mycobacterium tuberculosis supports protein tyrosine phosphorylation. Proc Natl Acad Sci USA.

[32] Chao JD, Wong D, Av-Gay Y (2014). Microbial protein-tyrosine kinases. J Biol Chem.

[33] Mattow J (2003). Comparative proteome analysis of culture supernatant proteins from virulent Mycobacterium tuberculosis H37Rv and attenuated M. bovis BCG Copenhagen. Electrophoresis.

[34] de Souza GA, Leversen NA, Malen H, Wiker HG (2011). Bacterial proteins with cleaved or uncleaved signal peptides of the general secretory pathway. J Proteomics.

[35] Malen H, Berven FS, Fladmark KE, Wiker HG (2007). Comprehensive analysis of exported proteins from Mycobacterium tuberculosis H37Rv. Proteomics.

[36] Weldingh K (1998). Two-dimensional electrophoresis for analysis of Mycobacterium tuberculosis culture filtrate and purification and characterization of six novel proteins. Infect Immun.

[37] Kyuma T, Kizaki H, Ryuno H, Sekimizu K, Kaito C (2015). 16S rRNA methyltransferase KsgA contributes to oxidative stress resistance and virulence in Staphylococcus aureus. Biochimie.

[38] Bitter W (2009). Systematic genetic nomenclature for type VII secretion systems. PLoS pathogens.

[39] Ates LS (2015). Essential Role of the ESX-5 Secretion System in Outer Membrane Permeability of Pathogenic Mycobacteria. PLoS Genet.

[40] Serafini A, Pisu D, Palu G, Rodriguez GM, Manganelli R (2013). The ESX-3 secretion system is necessary for iron and zinc homeostasis in Mycobacterium tuberculosis. PLoS One.

[41] Maciag A (2007). Global analysis of the Mycobacterium tuberculosis Zur (FurB) regulon. J Bacteriol.

[42] Rodriguez GM, Voskuil MI, Gold B, Schoolnik GK, Smith I (2002). ideR, An essential gene in mycobacterium tuberculosis: role of IdeR in iron-dependent gene expression, iron metabolism, and oxidative stress response. Infect Immun.

[43] Imlay JA, Chin SM, Linn S (1988). Toxic DNA damage by hydrogen peroxide through the Fenton reaction *in vivo* and *in vitro*. Science.

[44] Mehra A (2013). Mycobacterium tuberculosis type VII secreted effector EsxH targets host ESCRT to impair trafficking. PLoS pathogens.

[45] Av-Gay Y, Jamil S, Drews SJ (1999). Expression and characterization of the Mycobacterium tuberculosis serine/threonine protein kinase PknB. Infect Immun.

[46] Hildebrandt E, Fried VA (1989). Phosphoamino acid analysis of protein immobilized on polyvinylidene difluoride membrane. Anal Biochem.

