# Supplementary material for: Protein tyrosine kinase, PtkA, is required for Mycobacterium tuberculosis growth in macrophages
Source: Sci Rep. 2018 Jan 9;8:155. doi: 10.1038/s41598-017-18547-9 (PMC5760654; doi:10.1038/s41598-017-18547-9)
Supplement: Supplementary file 1 — Supplementary Information [file 41598_2017_18547_MOESM1_ESM.pdf]

Protein tyrosine kinase, PtkA, is required for *Mycobacterium tuberculosis* growth in macrophages

Dennis Wong, Wu Li, Joseph D. Chao, Peifu Zhou, Gagandeep Narula, Clement Tsui, Mary Ko, Jianping Xie, Carlos Martinez-Frailes and Yossef Av-Gay*

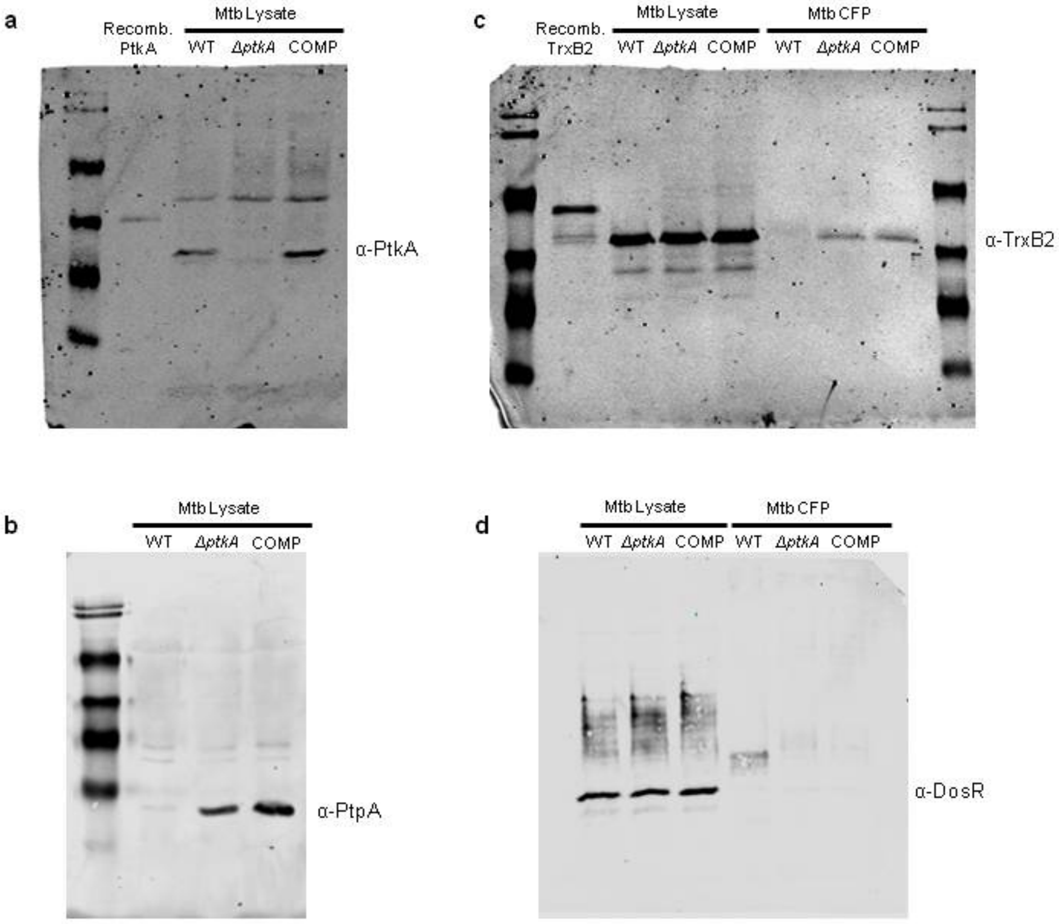

Supplementary Figure 1. Western analysis of protein expression. Western blot of PtkA (**a**), PtpA (**b**), TrxB2 (**c**), and DosR (**d**) from cellular lysate and culture filtrate fractions (where indicated) prepared from WT Mtb, Δ*ptkA* and complemented strains grown in Sauton's media. The cytoplasmic DosR protein was used as a control for cell lysis in the culture filtrate fraction. Recomb, His-tagged recombinant purified PtkA (5 ng) and TrxB2 (5 ng) were used as controls in (**a**) and (**c**), respectively.

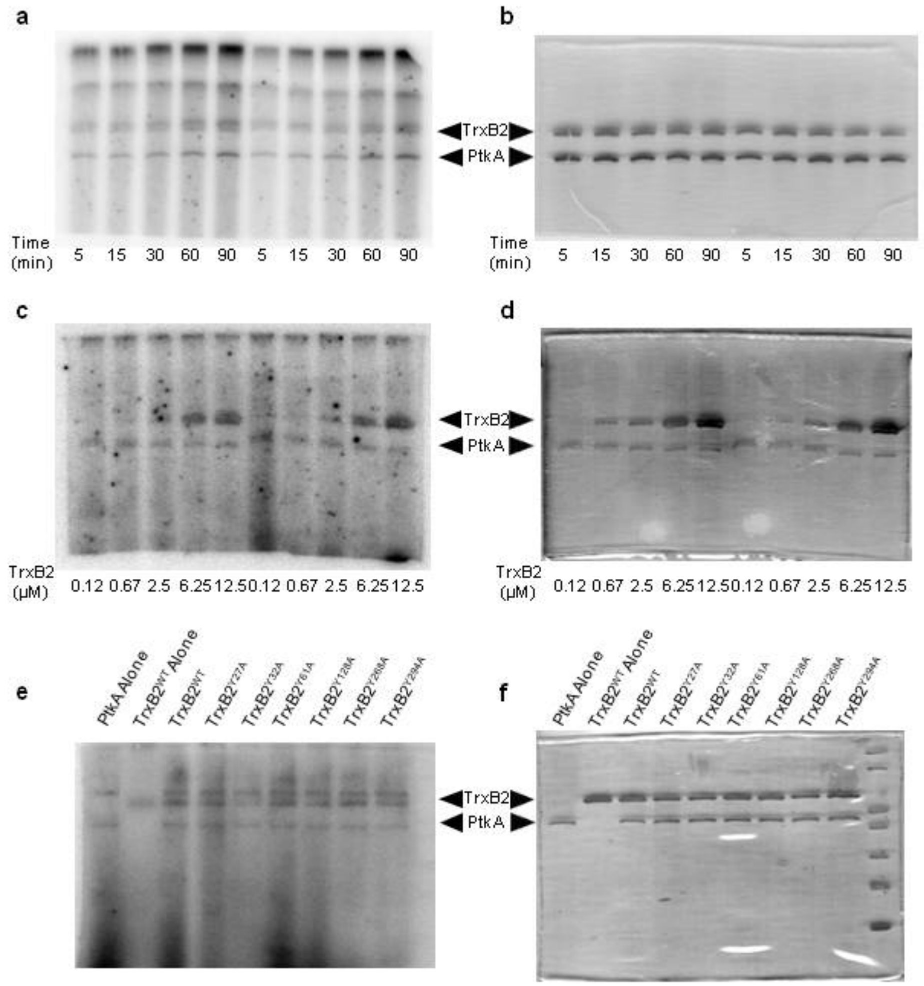

Supplementary Figure 2. PtkA dependent tyrosine phosphorylation of TrxB2. *In vitro* kinase assay demonstrating time-dependent (**a,b**) and dose-dependent (**c,d**) phosphorylation of TrxB2 by PtkA using [γ-^{32}P]ATP. Experiments were performed in triplicate; duplicates are shown. **e,f** *In vitro* kinase assays with [γ-^{32}P]ATP of PtkA-phosphorylated TrxB2 recombinant proteins with single amino acid Tyr-Ala point mutations. Autoradiography images shown on the left (**a**, **c**, **e**) and silver-stained (**b**) or coomasie blue-stained (**d**, **f**) SDS-PAGE on the right.

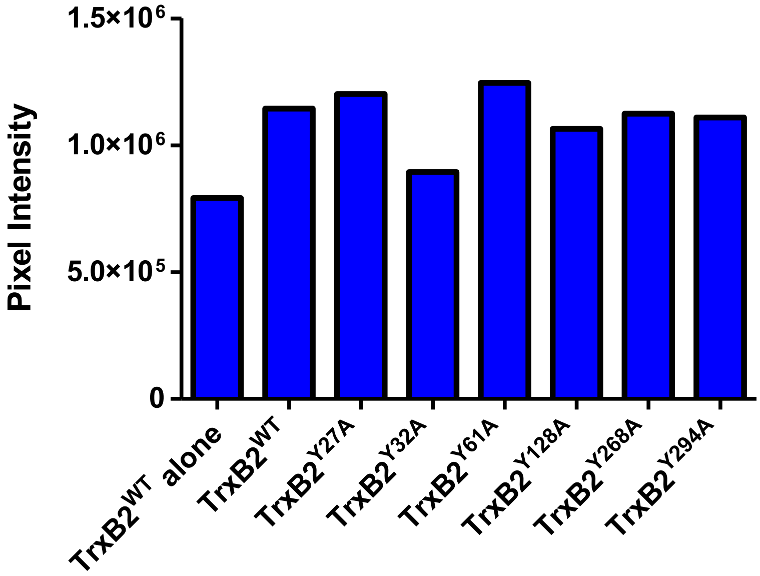

Supplementary Figure 3. Densitometric analysis of TrxB2 phosphorylation. The phosphorylation levels of the various TrxB2 recombinant proteins from Fig. 6d were analyzed by measuring the Integrated Density of the inverted bands using Photoshop.
